# Health promotion interventions in social economy companies in Flanders (Belgium)

**DOI:** 10.1186/s12889-015-2682-5

**Published:** 2016-01-05

**Authors:** Anne Hublet, Lea Maes, Jasmine Mommen, Benedicte Deforche, Ilse De Bourdeaudhuij

**Affiliations:** 1 Department of Public Health, Ghent University, De Pintelaan 185, 4K3, 9000 Ghent, Belgium; 2 Department of Human Biometry and Biomechanics, Vrije Universiteit Brussel, 1050 Brussels, Belgium; 3 Department of Movement and Sport Sciences, Ghent University, 9000 Ghent, Belgium

**Keywords:** Health promotion, Persons with disabilities, Supported employment

## Abstract

**Background:**

Disadvantaged groups are often not reached by mainstream health promotion interventions. Implementing health promotion (HP) interventions in social economy companies, can be an opportunity to reach those people. The implementation of these interventions in social economy companies was studied. Factors that could be related to the implementation of HP and being supportive towards implementation in the future, were investigated.

**Methods:**

An online, quantitative survey was sent to all 148 sheltered and social workshops in Flanders. In the questionnaire, the status of HP interventions and characteristics of the workshop were explored. Personal factors (such as attitudes towards HP, behavioural control, social norms and moral responsibility) were asked to the person responsible for implementation of HP interventions. Univariate and multivariate logistic regressions were performed.

**Results:**

Respondents of 88 workshops completed the questionnaire. Almost 60 % of the workshops implemented environmental or policy interventions. Having a positive attitude towards HP, being more morally responsible, and having the subjective norm that employees are positive towards health promotion at work, were related to being more supportive towards the implementation of HP in the univariate analyses. Only attitude stayed significantly related to being more supportive towards the implementation of HP in the multivariate analyses.

**Conclusions:**

Sheltered and social workshops are open to HP interventions, but more can be done to optimize the implementation. To persuade persons responsible for the implementation of HP to invest more in HP, changing attitudes concerning the benefits of health promotion for the employee and the company, is an important strategy.

## Background

People with intellectual and physical disabilities, or psychiatric problems are more likely to be at risk of an unhealthy lifestyle. They are more likely to have obesity and to be less physically active [1–3], more at risk for depression [4] and more likely to be a smoker [2]. Research concerning Health Promotion (HP) for people with disabilities or psychiatric problems is underdeveloped and this population is hard to reach by mainstream HP initiatives [5, 6]. Therefore, HP for people with disabilities should be implemented in their natural settings such as in supported living facilities and day care centers [5].

One of these natural settings can be the social economy companies. These companies “seek to serve the community’s interest rather than profit maximization” [7]. They employ society’s most fragile members, and in that way, contribute to social cohesion, employment and the reduction of inequalities. Also in Flanders-Belgium, social economy companies employ a diverse group of people who are (yet) unable to work in the regular economy. Most of them have a low educational level and are living in precarious life conditions [8].

The focus of this study is on HP interventions in social economy companies, defined as the promotion of healthy nutrition, physical activity, better mental health, and the prevention of smoking and alcohol (ab)use, themes also found in the health targets of the Flemish Government [9]. An unhealthy lifestyle is one of the major risk factors for noncommunicable diseases (e.g. cardiovascular diseases, cancers) [10]. Employees with an unhealthy lifestyle are more likely to be absent due to sickness, the length of their absenteeism is longer and their productivity lower [11].

Research has shown that HP at the workplace has positive effects on the health of employees and has advantages for the company. Verweij et al. found evidence that physical activity and nutrition interventions at the workplace had a positive effect on body weight, BMI and body fat [12]. While for the company, the promotion of health behaviour had a positive influence on absenteeism, job performance [13], productivity and presenteeism [14].

But not all interventions are equally effective. Interventions with an environmental component that include environmental modifications were found to be more effective, than those without environmental changes [12, 15]. Also policy measures (e.g. smoking or alcohol regulations) had more chance to have long-lasting results [16]. These interventions (further called ‘environmental HP interventions’) influence both the conscious and unconscious behaviour and habits of the employees [17]. Other interventions such as temporary educational group sessions, individual counseling and short running actions were less effective in the long-term [18].

In order to promote HP interventions in social economy companies, it is important to get insight into the determinants that are related to the implementation of these interventions. These factors can be characteristics of the company (such as size and sector), but also individual factors of the person responsible for the implementation of HP interventions. The Theory of Planned Behaviour [19] can be used to explain the implementation of HP interventions at an individual level. In this model, the three constructs ‘attitude’, ‘subjective norm’ and ‘behavioural control’, predict the intention to implement HP interventions, while the intention predicts the implementation. Besides these three ‘classical’ constructs, Ajzen [20] argued that in some contexts, personal feelings of moral responsibility could add power to the model.

In this study, three aims were formulated. The first aim was to investigate the current status of the implementation of HP interventions in social economy companies in Flanders- Belgium. The second aim was to investigate which characteristics of the company and factors of the person responsible for implementing HP, were related to the implementation of environmental HP interventions. The third aim was to investigate which characteristics of the company and which personal factors of the person responsible for implementing HP, were related to being supportive towards investing more in HP in the future.

## Methods

### Design

An online, quantitative survey was organized. An email with an invitation to participate was sent to all sheltered and social workshops in Flanders (*n* = 148). The social economy in Flanders comprises four types of companies, each with their own target population [8]. In this study, two types were included which employ the largest group of disadvantaged people. *Sheltered workshops* employ mainly people with disabilities (intellectual and physical). *Social workshops* provide employment to people with physical, social or psychological problems (e.g. people with psychiatric problems, people reintegrating into society after prison, immigrants). The two excluded types of social economy companies were the local service economy who employ older people who are already long-term unemployed, and the insertion companies who provide a job for people with a low education level together with a history of long-term unemployment. The email-list was provided by the umbrella-organization for the social economy (CollondSe). The person who would normally be responsible for implementing HP interventions, completed the questionnaire. After two weeks, a reminder was sent. The study was executed from February to April 2013. The study was approved by the ethical committee of the Ghent University Hospital (2013/076). The respondents gave their informed consent for participation in the study by clicking on the link to the questionnaire.

### Questionnaire

In the first part of the questionnaire, the current status of HP interventions and the characteristics of the workshop were questioned. The current status of HP was assessed by: “Does the company organize HP actions, besides the obligatory smoking ban at the workplace? Yes or no” and “If so, for which themes and how did the company organize HP?”. Examples were given for each HP action to make sure respondents knew what is understood under HP. The examples were: policy changes (e.g. an alcohol ban during lunch), environmental changes (e.g. providing fruit for free), education in groups (e.g. group session on healthy food), individual guidance (e.g. counseling at the social department), and short running actions (e.g. a smoke-free day). The themes were: nutrition, physical activity, smoking, alcohol use and mental health. Five new variables were constructed by recoding per theme the options ‘policy changes’ or ‘environmental changes’. The sum of these five variables was made and recoded into a new variable with categories ‘implemented an environmental intervention’ and ‘no environmental intervention implemented’.

Three characteristics of the workshop were assessed. First, the type of workshop (sheltered or social workshops) was asked. Second, the size of the workshop was asked and recoded into small (less than 49), medium (between 50 and 249) and large (250 employees or more) companies. Finally, the economical sector was asked including: primary (agriculture, retrieval of raw materials), secondary (industrial sector), tertiary (supplies commercial services) and quaternary sector (not-commercial service sector e.g. hospitals, education, social work, cultural sector).

In the second part, the personal opinion of the respondent about HP was questioned. This respondent was the person responsible for implementing HP in the company or the person that could have that task (if no HP was already implemented).

Being supportive towards implementing HP in the future, was asked by the question: ‘Are you a supporter to invest more in HP at your company in the future?’. A 5-point Likert scale was used ranged from ‘1-totally disagree’ to ‘5-totally agree’. Because of a skewed distribution, the variable was dichotomized into 0 ‘no supporter or neutral’ (scores 1–3) and 1 ‘being a supporter’ (scores 4 and 5).

A second question assessed the perception of the respondent on the statement if employees with a disability benefit from health promotion initiatives. The answer possibilities to that question were: 1) yes and there are enough suitable interventions for this specific group, 2) yes but the existing interventions are not adapted to people with a disability and therefore the results are limited, 3) no because the target group is not open for health messages, 4) no because the health and social issues of the target group are too big for the means that are available in the company.

The questions about the personal factors, derived from the Theory of Planned Behaviour, were based on the questionnaire developed by Downey and Sharp [21]. To be in line with the other questions in the questionnaire, all answers were rated on a 5-point Likert scale, instead of the 7-point Likert scale used in the original questionnaire [21]. The questionnaire was adapted to the Belgian situation, such as the inclusion of the trade union in the subjective norm scale and the exclusion of questions about discretionary spending on health care. Clarity of the questions and exhaustiveness of the questionnaire were tested in people working in the umbrella organization of the workplaces and some employees of the social department of the workplaces.

Attitude was measured by two constructs: behavioural beliefs (the perception of the respondent concerning the benefits of HP, e.g. investing more in HP will increase the moral of employees) and outcome evaluations (the importance the respondent gives to these benefits, e.g. trying to improve employees’ morale is desirable). Five different beliefs and their accompanying evaluations were assessed. The attitude-score was calculated by multiplying beliefs with the outcome evaluation and dividing them by 5 (see Table 1 for the 5 attitudes asked). The higher the attitude-score, the more likely the respondent beliefs that HP is beneficial and that the outcome is desirable. A total attitude-score was calculated by taking the mean of the 5 attitudes. The Cronbach alpha for the scale was 0.82.

**Table 1:** Mean and standard deviation of the personal factors of the respondent responsible for implementing HP in the company

Attitude:	Belief of HP outcomes (with ‘1-very unlikely’ to ‘5-very likely’) * Outcome evaluation (with ‘1-very undesirable’ to ‘5-very desirable’)	Mean (standard deviation)
	- HP increases the moral of employees (0.20–5)	2.72 (1.04)
	- HP leads to an increase in productivity (0.20–5)	2.64 (1.19)
	- HP results in a longer life (0.20–5)	2.28 (1.01)
	- HP leads to a decrease in absenteeism (0.20–5)	3.16 (1.11)
	- HP results in a decrease of turnover (0.20–5)	2.17 (1.07)
	Total attitude scale (0.20–5)	2.60 (0.82)
Behavioural control:	How much control do you have on …(with ‘1–no control’ to ‘5–total control’)	
	- the implementation of HP activities (1–5)	3.54 (0.96)
	- resources such as personnel and time (1–5)	2.69 (1.24)
	How extensive is your participation in securing budgets? (1–5)	2.77 (1.30)
	Total control scale (1–5)	3.01 (1.04)
Subjective norm:	Normative beliefs (‘How likely is it that following persons believe that you should invest in HP’ with ‘1–very unlikely to approve’ to ‘5–very likely to approve’) * Motivation to comply (‘How important is the opinion of following persons’ with ‘1–very unimportant’ to ‘5–very important’)	
	- the person or committee above you (0.20–5)	2.40 (0.93)
	- colleagues (0.20–5)	2.67 (0.91)
	- clients (0.20–5)	1.38 (0.89)
	- co-owners (0.20–5)	1.39 (0.95)
	- employees (0.20–5)	2.38 (0.96)
	- other companies (0.20–5)	0.96 (0.80)
	- the community (0.20–5)	1.78 (1.01)
	- the trade unions (0.20–5)	2.08 (1.19)
	Total subjective norm scale (0.20–5)	1.93 (0.66)
Moral responsibility:	How much do you agree with following statements? (with ‘1–very disagree’ to ‘5–very agree’)	
	- The benefits of HP exceed the costs of HP (1–5)	3.39 (0.85)
	- I have the moral obligation to ameliorate the health of my employees (obligation) (1–5)	3.63 (0.94)
	- As employees are spending a long time during the day in my company, it is fair that I invest in their health behaviour (fairness). (1–5)	3.67 (0.89)
	Total moral responsibility scale (1–5)	3.56 (0.67)

Behavioural control was measured using three questions on control over implementation, resources and budgets. For the total scale, the mean of the three questions was calculated. The Cronbach alpha for the scale was 0.87.

The subjective norm was measured by two constructs: normative beliefs (the perception of the (dis)approval of a reference group concerning HP, e.g. how likely is it that your colleagues believe that you must invest resources in HP?) and the motivation to comply (the importance of this reference group for the respondent, e.g. how much do you care whether your colleagues approve that you invest in HP?). Normative beliefs and motivation to comply were assessed concerning eight reference-groups: the seven reference-groups from the questionnaire of Downey and Sharp [21], plus the reference-group ‘trade union’ (Table 1). As with the attitude scale, the scores of the normative beliefs and their accompanying motivation to comply were multiplied and divided by 5. The higher the score on the subjective norm, the more likely the respondent believes that HP should be implemented according to a relevant reference-group.’ A total score was calculated by taking the mean. The Cronbach alpha for this scale was 0.77.

Moral responsibility was measured using a scale developed by Hart [22]. Three dimensions of moral responsibility were included (see Table 1 for the themes). The mean was used as total score on moral responsibility. The Cronbach alpha for this scale was 0.60.

### Data analysis

To investigate the current status of the implementation of HP interventions (aim 1), percentages were given of the currently implemented HP themes and actions. Chi^2^-test were used to analyze if these results differed by the characteristics of the workshop.

To investigate which characteristics of the workshop and individual factors of the respondent were related to the implementation of environmental HP (aim 2) and to being supportive towards implementing HP in the future (aim 3), univariate logistic regressions were used. A multivariate logistic regression was performed with all significant factors from the univariate analyses.

SPSS 21 was used to analyze the data. *P*-values lower than 0.05 were considered to be statistically significant.

## Results

### Descriptives

Eighty-two workshops completed the online questionnaire (response rate 55.4 %), of which 65.9 % social workshops and 34.1 % sheltered workshops. Half of them were medium-sized (48.1 %), followed by large-sized (27.8 %) and small companies (24.1 %). Concerning the economical sector, 11.2 % could be categorized into the primary sector, 31.2 % into the secondary sector, 40 % into the tertiary sector and 17.5 % into the quaternary sector.

The means and standard deviations of the personal factors of the respondent can be found in Table 1. Of the 82 respondents, 24 were directors of the company, 32 were working at the social department, 15 were working on the personnel department, 7 were department coordinators and 4 were prevention advisors.

### Aim 1: Current status of HP in sheltered and social workshops

Of the workshops, 64.6 % indicated that they organized HP. The theme that was most frequently chosen to work on in a HP intervention was alcohol use (58.5 %), followed by nutrition (50 %), mental health (37.8 %), tobacco use (36.6 %) and physical activity (28 %). The kind of interventions that were already implemented were most policy changes (43.1 %) and individual guidance (43.1 %), followed by education in group (31.5 %), environmental changes (26.2 %) and short running actions (11.5 %). The information about which actions per theme were implemented, can be found in Table 2.

**Table 2:** Cross tabulation of the 5 HP themes with the HP actions that were implemented in the 82 workshops

	Changes in policy	Changes in environment	Education in group	Individual guidance	Short running actions
Nutrition	17	16	22	23	4
Physical activity	6	3	11	13	7
Tobacco	18	5	9	18	1
Alcohol use	37	3	15	34	2
Mental health	4	3	9	27	3

No significant differences in size of the company (chi^2^ = 0.997, df = 2, *p* = 0.607), type of workshop (chi^2^ = 0.002, df = 1, *p* = 0.962) or sector (chi^2^ = 4.985, df = 3, *p* = 0.173) were found between those who had implemented HP and those not.

Also, no significant differences were found between the themes and kind of interventions by type of workshop or by sector. One significant difference was found by size. Large workshops were more likely to facilitate group education (54.8 %), compared with medium-sized (36.8 %) and small workshops (15.8 %) (chi^2^ = 6.592, df = 2, *p* = 0.037).

Environmental HP interventions were more commonly implemented for alcohol (45.1 % of the workshops), followed by nutrition (31.7 %), and tobacco (23.2 %). Only 9.8 % of the workshops implemented an environmental intervention concerning physical activity and 7.3 % concerning mental health.

Only twelve percent of the respondents indicated that employees with a disability benefit from HP initiatives and that there are suitable interventions available for this specific group. More than half of the respondents (55 %) answered that employees with a disability could benefit from HP interventions but that there are no interventions available that are adapted to the target group. The other 33 % of the respondents answered that employees with a disability do not benefit from HP initiatives: 26 % indicated that the target group is not open for health messages, and 7 % indicated that the health and social issues of the target group are too big for the means that are available in the company.

### Aim 2: Factors of implementing environmental HP interventions

Almost 60 % (59.8 %) of the workshops had one or more environmental HP intervention implemented. In univariate logistic regressions, none of the characteristics of the workshop and none of the personal factors of the respondents were related to having an environmental intervention implemented.

### Aim 3: Factors of being supportive towards investing more in HP in the future

Half of the respondents (50 %) were supportive towards investing more in HP in the future. In Table 3, the results of the univariate logistic regressions with the characteristics of the workshop and the personal factors, can be found.

**Table 3:** Univariate and multivarariate logistic regressions with being a supporter to implement more HP as dependent variable and characteristics of the company and personal factors of the respondent as independent variables

	Univariate analyses		Multivariate analyses	
	OR^a^	95 % CI^b^	OR^a^	95 % CI^b^
Characteristics of the workshop				
Company size (base = large)				
Small	1.00	0.26 – 3.82		
medium-sized	2.75	0.84 – 8.98		
Sector (base = quaternary)				
Primary	1.00	0.17 – 5.99		
Secondary	0.82	0.19 – 3.43		
Tertiary	1.14	0.30 – 4.36		
Program (base = social workshop)				
sheltered workshop	1.14	0.42 – 3.06		
Personal factors of the respondent				
attitude: increase moral employees	2.64	1.45 – 4.81**		
attitude: increase productivity	2.10	1.289 – 3.41**		
attitude: increase life years	1.84	1.08 – 3.13*		
attitude: decrease absenteeism	2.28	1.35 – 3.85**		
attitude: decrease turn over	2.15	1.25 – 3.69**		
Attitude total	5.15	2.06 – 12.88***	3.82	1.41–10.36***
subjective norm: person or committee above you	1.06	0.64 – 1.78		
subjective norm: colleagues	1.48	0.86 – 2.55		
subjective norm: clients	1.25	0.72 – 2.17		
subjective norm: co-owners	1.81	0.99 – 3.33		
subjective norm: employees	1.87	1.07 – 3.25*	1.42	0.74 – 2.71
subjective norm: other companies	1.15	0.62 – 2.15		
subjective norm: the community	0.95	0.59 – 1.54		
subjective norm: the trade unions	0.98	0.65 – 1.47		
Subjective norm total	1.52	0.70 – 3.27		
control over implementation	1.32	0.80 – 2.18		
control over resources	1.11	0.76 – 1.63		
control over budget	0.93	0.64 – 1.33		
Control total	1.11	0.71 – 1.74		
moral: benefits versus the costs	2.17	1.15 – 4.10*	1.27	0.59 – 2.73
moral: obligation	1.78	1.03 – 3.05*	1.36	0.71 – 2.63
moral: fairness and justice	1.70	0.96 – 3.02		
Moral responsibility total	3.29	1.40 – 7.73**		

^a^ Odds Ratio; ^b^ 95 % Confidence Interval; *p < 0.05, **p < 0.01, ***p < 0.001

None of the characteristics of the workshop were related to being supportive towards more HP in the future. All attitude variables were positively related to being supportive. Respondents scoring one point higher on the attitude scale were even 5 times (OR = 5.15) more likely to being supportive to more HP than those scoring one point less. Respondents believing that employees are expecting that the workshop invest in HP, were more likely to be a supporter of more HP in the future. Respondents agreeing that the benefits of HP exceeds the costs, and those who had the belief that HP is an obligation, were more likely to be supportive. Also, the total moral responsibility scale was positively related to being supportive towards more HP.

A multivariate logistic regression was performed with all significant results. As all attitudes and the total scale was significant related to the dependent variable, the total scale was chosen to be included in the analyses (Table 3). Only the attitude-scale stayed significantly related to being supportive towards more HP in the workshop in the future.

## Discussion

Sheltered and social workshops in Flanders (Belgium) employ people from disadvantaged groups who are less likely to be reached by mainstream HP interventions. Therefore, these companies might be a good channel to provide HP in disadvantaged people. In this study, the current status of HP interventions in these companies and factors related to the implementation were studied.

Companies can organize HP in different ways, but environmental and policy changes are seen as most effective and long-lasting [12, 15, 16]. In this study, almost 60 % of the workshops had an environmental or policy HP intervention implemented. Besides environmental and policy HP interventions, individual HP strategies can be used to increase the knowledge and positive attitudes concerning a healthy lifestyle, or to improve the self-efficacy to perform the healthy behaviour [23]. Workshops also invest in these strategies: 40 % gave individual guidance to their employees and one third organized educational group sessions.

In 2012, the Flemish Institute for Health Promotion and Disease Prevention organized a survey on health indicators in general Flemish companies [24]. In their report, percentages of HP ranged from 15 % for nutritional topics to 40 % for tobacco interventions. Although they used other measurements to assess HP, our results indicate that workshops have almost equal or more attention to HP themes compared with normal economy companies. The daily confrontation with health-related problems of their employees, such as high sickness rates due to unhealthy lifestyle, and alcohol and tobacco dependency problems, can possibly have urged workshops to invest in HP.

In low-wage industries, Hannon et al. showed that larger companies (up to 250 employees) were more likely to implement workplace HP (WHP) compared with smaller companies (between 100–249 employees) [25]. Also in the Flemish report on health indicators, the same was observed [24]. In addition, Flemish companies working in the tertiary sector, were less likely to have HP implemented compared with companies from the secondary and quaternary sector (primary sector not included in this study). But in the present study, none of these characteristics were related to having an environmental HP intervention or to being supportive towards investing in HP in the future.

According to the Theory of Planned Behaviour, this study found that having a positive attitude towards HP was related to being a supporter of investing more in HP in the future. Also, in the study of Downey & Sharp in normal economy companies, a positive attitude was the best predictor of the intention to implement WHP [21]. They also found that the perception of control over resources and budget in human resource managers and moral responsibility in general managers predicted the intention to implement WHP. In our study, control was not related to being a supporter of investing more in HP. Moral responsibility was significantly related in the univariate analyses but the significance disappeared when controlling for the other significant factors. Subjective norm was not a significant predictor in both studies.

However, none of these factors were related to the current implementation of HP in the workshop. This can partly be explained by the intention-behaviour gap. Some barriers may exist between having a positive intention to invest in HP and the actual behaviour of implementing HP. Known barriers for the implementation of HP are a lack of time and logistical challenges [26]. Also in workshops, these barriers may exist as mostly only one or two persons are responsible for the implementation of HP. Another barrier can be the lack of tailored interventions for this special group [5]. In our study, 55 % of the respondents indicates that there are no suitable interventions for the target group. More research is needed to find better individual predictors of (the intention to invest in) the implementation of HP at the workplace.

But also methodological problems can explain this result. The question we asked was if the company had already implemented HP interventions and not if the respondent had experience in implementing HP in the company. Therefore, other persons could have been responsible for the implementation of the current HP interventions. Also, no questions were asked about the socio-demographic characteristics (such as age and gender) and the respondents’ knowledge on and skills for implementing HP interventions, while these factors can be important in predicting the implementation of HP at the workplace. If the respondents have limited knowledge on HP and have not much expertise in implementing HP, they will be less aware about possible HP actions and their implementation. In addition, we have no information about details and quality of specific interventions that were implemented, such as duration of intervention, type of education sessions and dissemination of the intervention. This lack of information can be seen as a limitation of this study.

Another limitation is the low response rate and relatively small sample size. Only 55 % of the workshops participated in the study, which limits the generalization of the results. Of all social workshops, 57.4 % participated in the study compared to 51.9 % of all sheltered workshops. This low response may be associated with the reconstruction of sheltered and social workshops in 2013, leading to an overload of work and insecurities for the companies. Therefore, it may be that they were less motivated to cooperate to this project.

Finally, only univariate analyses could be performed to study aim 2 as none of the included variables were significantly related to implementation of HP at the workplace.

### Implications

Despite these limitations, some implications can be formulated. To persuade HP implementers in workshops to invest more in HP in the future, disseminators of HP interventions have to influence the attitudes towards HP in these implementers. This can be done by increasing the knowledge of implementers about HP interventions in workshops, to show them the advantages of HP interventions, and to discuss negative consequences of the absence of HP in this specific group of employees [23]. However to our knowledge, until now, the effectiveness of HP interventions for disadvantaged groups in the setting of workshops is not studied. Therefore, research is needed on the effectiveness of HP on e.g. decreasing absenteeism and increasing the moral of employees working in workshops. Also cost-effectiveness studies are needed to prove that the benefits of HP outweigh the costs.

Besides working on the attitude of the implementers, it is also important to increase their skills to plan, implement and evaluate HP initiatives. In most workshops, only one or two persons are responsible for the implementation of HP. Therefore, tools should be developed to help them and guide them through the different stages of implementation. An important topic in these tools is how to make the HP project a team effort instead of the project of one or two individuals.

But to have sufficient effect, interventions should be tailored to the specific target group and setting. As HP in people with disabilities is still underdeveloped [5], workshops are using HP interventions that are not adapted to the needs of these employees or to the setting of workshops. One of the elements of structural feasibility for the implementation of HP in workplaces are the literacy levels, which can be low in workshops [27]. Therefore, interventions such as choice architecture, that alter the properties or placements of objects in the workplace with the intention to change health-related behaviour, should be investigated as these interventions require minimal conscious engagement, and can change the behaviour of many people simultaneously [17]. As 55 % of the persons responsible for implementing HP indicated that existing interventions are not adapted to people with a disability, more research is needed on which HP interventions are suitable for people with disabilities (in terms of attainability) and which HP interventions are effective in this target group (in terms of behavioural change and health outcome).

## Conclusion

Although this study described the specific situation of sheltered and social workshops in Flanders (Belgium), the results of this study could be used to optimize dissemination of HP in companies working with employees of so-called disadvantaged groups. Creating positive attitudes towards HP could make the implementer more supportive towards investing in HP in the future.

## Abbreviations

HP: Health Promotion
WHP: Workplace Health Promotion
